# Torsed gangrenous Meckel’s diverticulum causing gangrenous ileal segment: A rare case report of small bowel obstruction in children

**DOI:** 10.1016/j.amsu.2021.102723

**Published:** 2021-08-17

**Authors:** Saroj Kumar Jha, Sharmila Ghimire, Dinesh Prasad Koirala

**Affiliations:** a Maharajgunj Medical Campus, Institute of Medicine, 44600, Kathmandu, Nepal; b Pediatric Surgery Unit, Tribhuvan University Teaching Hospital, Institute of Medicine, 44600, Kathmandu, Nepal

**Keywords:** Meckel's diverticulum, Gangrene, Bowel obstruction

## Abstract

**Introduction:**

Meckel's diverticulum (MD) is the most common congenital anomaly of the gastrointestinal system. It is caused by an incomplete obliteration of the vitelline duct. Rarely, it can present with complications like torsion and gangrene formation.

**Case presentation:**

A 13-year previously healthy girl presented with sudden onset periumbilical pain and bilious vomiting who was subsequently diagnosed with Meckel's diverticulum. Intraoperatively, torsed gangrenous diverticulum forming band adhesion was found. Resection of Meckel's diverticulum along with gangrenous ileal segment followed by ileoileal anastomosis was done.

**Discussion:**

Axial torsion of Meckel's diverticulum with gangrene formation is a rare occurrence. Mesodiverticular band adhesion along with herniation of small bowel segments under it endangers viability of herniating segments. Preoperative diagnosis of complicated MD is difficult as it mimics other common acute abdominal conditions. CT scan and enteroclysis are imaging modalities of choice. Surgical resection of MD along with resection and anastomosis of gangrenous bowel segment results in complete cure.

**Conclusion:**

Meckel's diverticulum with complications should be kept in the differential of acute abdominal conditions presenting with atypical symptoms. Surgical resection ensures complete cure.

## Highlights

• Meckel's diverticulum (MD) is the commonest congenital anomaly of the gastrointestinal system.
• Torsion of MD resulting in gangrene is a rare cause of small bowel obstruction.
• Preoperative diagnosis of complicated MD is difficult, but CT scan may be of some help.
• Surgical resection of gangrenous bowel MD remains standard choice of management.

## 1 Introduction

Meckel's diverticulum (MD) is the most common congenital anomaly of the gastrointestinal system, affecting 2% of the general population [1]. The majority of MD cases are asymptomatic. Just 4% of Meckel's diverticulum patients experience complications such as bleeding, perforation, inflammation, or intestinal obstruction [2]. Various mechanisms, such as intussusception of an inverted Meckel's diverticulum, volvulus, Littre's hernia, axial torsion, and internal herniation of the small bowel under the mesodiverticular band, cause intestinal obstruction [3]. The most uncommon complications recorded in the literature are axial torsion and gangrene formation [4]. We report a very rare case of intestinal obstruction caused by axial torsion of Meckel's diverticulum with gangrene formation, in line with SCARE checklist [5].

## 2 Presentation of case

**Fig. 1:**
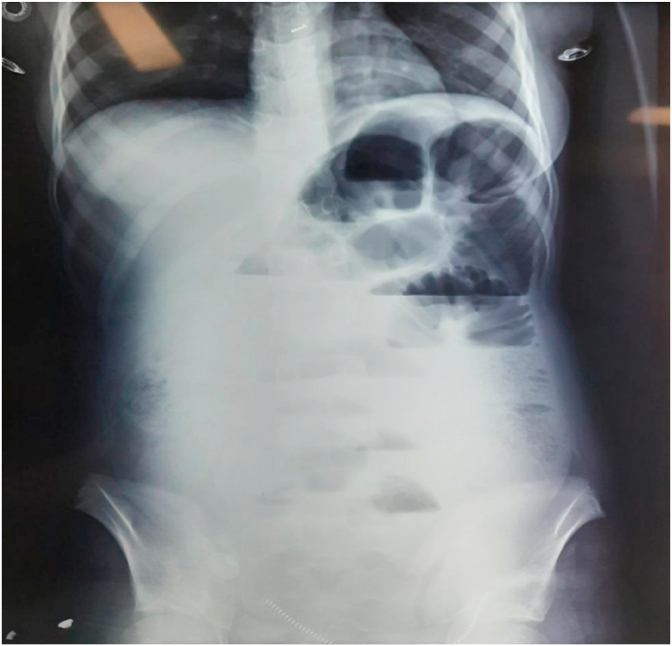
Plane X-ray showing small bowel obstruction.

A 13 years female presented with a 3 days history of abdominal pain. Pain was sudden onset, dull, moderate to severe, non-radiating, initially at periumbilical and hypogastric region which later generalized, and was associated with non-projectile, bilious vomiting of 2 episodes, and abdominal distension for 3 days. She was not able to pass stool or flatus for 3 days. She had no similar episodes in the past. There was no history of drug allergies. On examination, she was anxious, afebrile, dehydrated, tachycardic with blood pressure of 110/60 mmHg. There was mild distension and tenderness over lower abdomen along with rebound tenderness and guarding. Bowel sound was decreased. Complete blood count showed elevated white cell count and biochemical studies were within normal limits. Plain X-ray of the abdomen showed dilated small bowel loops with multiple air-fluid levels [Fig. 1]. USG abdomen showed slightly dilated bowel loops with interbowel loop ascites.

**Fig. 2:**
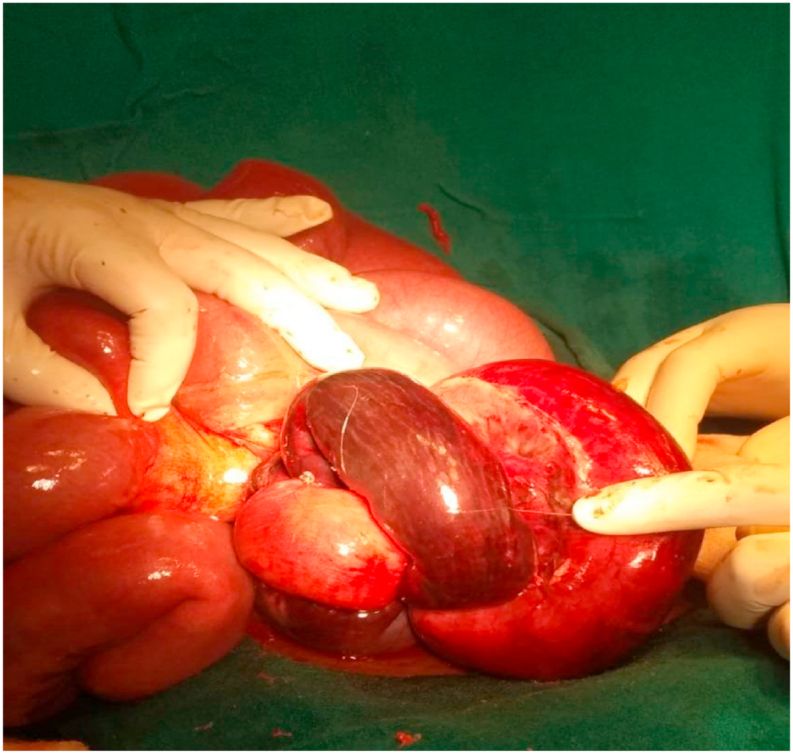
Gangrenous Meckel's diverticulum forming loop with band adhesion.

**Fig. 3:**
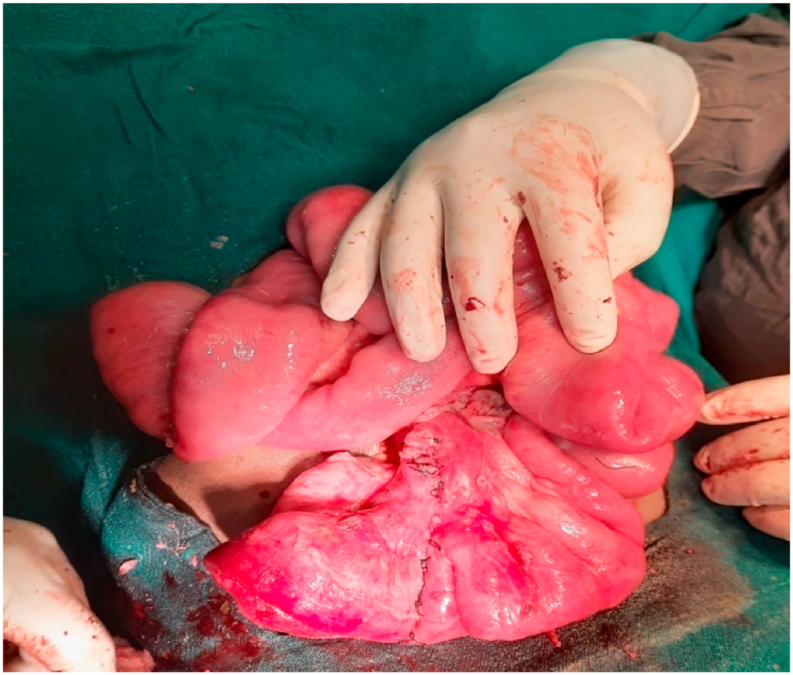
End-to-end ileoileal anastomosis.

With the clinical diagnosis of acute intestinal obstruction, urgent exploratory laparotomy was performed which revealed gangrenous Meckel's diverticulum of approximately 10 cm. It was swollen and twisted at its base with a width of 2 cm. Distal end of Meckel's diverticulum formed band adhesion with the mesentery of the adjacent ileum [Fig. 2]. Approximately 15 cm of adjacent ileal segment, 30 cm proximal to ileocaecal junction was also gangrenous. Distal segment of the ileum was herniating into closed space while proximal bowel loops were dilated with normal color. The diverticular band was divided and detorsion of Meckel's diverticulum was done. Herniating segments of ileum were reduced. Resection of gangrenous Meckel's diverticulum and segment of ileum was done along with end to end ileoileal anastomosis [Fig. 3]. Appendectomy was performed in the same setting. Surgery was performed by pediatric surgeon with superspecialization in field of pediatric surgery. Post operative period was uneventful and the patient was discharged home with analgesics after 14 days of hospital stay. Follow-up visit at 1 month and 6 month was uneventful. Patient was satisfied with the outcome of surgery.

## 3 Discussion

Meckel's Diverticulum was first described by Hildanus in 1598, and Johann Friedrich Meckel mentioned it in 1809, establishing its embryological origin [6]. It is a true diverticulum because it consists of all layers of the intestinal wall. It occurs when the omphalomesenteric duct, which connects the primitive gut to the yolk sac, is not fully obliterated during the seventh week of pregnancy [7].

The clinical presentation of MD can vary with little specificity. The ‘rule of 2's' has been observed in Meckel's diverticulum. The diverticulum may be 2 inches long and 2 feet from the ileocaecal valve, occur in 2% of the population, usually present in the first 2 years of life and twice as commonly in men as women [8]. Most cases of MD are asymptomatic. The two most common presentations in children are bleeding and intestinal obstruction which is seen in 25–50% and 25% of children respectively. While in adults, bleeding is the most common complication [9]. When intestinal obstruction does occur, intussusception or invagination with MD as the lead point is commonly implicated. A study conducted by Mares AJ et al. found that enterolith or bezoars lodged in the diverticulum in a Y-shaped “pantaloon” fashion is another uncommon cause for intestinal obstruction [10].

Gangrene of MD secondary to axial torsion is one of the rarest complications of MD and only few cases have been reported previously. The exact mechanism for torsion of MD around its narrow base is unclear; however some explanations have been put forward. Axial torsion of MD around its base and consequent gangrene formation has been related to the formation of omphalomesenteric or mesodiverticular bands attaching MD to umbilicus or ileal mesentery, respectively. The mesodiverticular band establishes an axis for diverticular torsion and also creates the underlying pathway for bowel to herniate [11]. In our case, loops of terminal ileum herniated through the passage created by MD and mesodiverticular band. During the event of herniation of the portion of terminal ileum in the loop of Meckel's diverticulum, it might have caused axial rotation of Meckel's diverticulum and thus gangrene formation [12]. The anatomical configuration, especially the length of diverticulum and its base diameter are important predisposing factors for torsion development. Elongated diverticulum (10 cm long in our case) with a narrow base (2 cm in our case), is more likely to result in torsion than short diverticulum with broad base [13]. Herniation often leads to bowel obstruction with dilatation of proximal bowel loops. In our case, there was the coexistence of gangrenous MD and its loop-forming mechanism of obstruction leading to gangrene of the proximal ileal segment. Bowel herniation in a loop formed by MD can be detrimental if left untreated as the bowel wall becomes edematous with decreased perfusion, leading to gangrene formation of herniated bowel loops, as in our case.

Diagnosis of symptomatic Meckel's diverticulum possesses special difficulty due to its clinical resemblance with other more common acute intra abdominal conditions e.g. appendicitis, peptic ulcer disease, IBD, other causes of small bowel obstruction. Plain radiographs may reveal associated small bowel obstruction and the presence of gas in the diverticulum or a gas–fluid level [14]. Enteroclysis has been shown to be more sensitive than regular barium examination [15]. Ultrasonography may indicate a tubular diverticulum distended with fluid in a site distant from the cecum, invagination, segmental thickening of the bowel walls, swelling of the diverticular wall, and pelvic abscess, albeit it is not precise enough to image this disease [16]. It is difficult to differentiate Meckel's diverticulum from normal small intestine in uncomplicated cases on CT scan. CT scan findings differ depending upon the complications associated with Meckel's diverticulum [17]. With a sensitivity and specificity of 85% and 95%, respectively, the 99mTc-pertechnetate scan (uptake by ectopic mucosa and identifying the location of gastrointestinal bleeding) is the most well-established approach for detecting Meckel's diverticulum [18]. However, diagnostic laparoscopy always remains the final pathway for the diagnosis of MD and its complications.

The gold standard of therapy for symptomatic MD is surgical resection. Surgical options include simple diverticulectomy or wedge resection or segmental resection. Ileal resection, as in our case, is done in MD complicated with gangrene formation of diverticulum or adjacent ileum. To avoid future morbidities, Meckel's diverticulum should be removed in asymptomatic children who are discovered accidently during abdominal exploration, as well as in situations when a concurrent mesodiverticular band is present [19]. However, decision regarding excision of asymptomatic MD is still debatable.

Cullen et al. reported postoperative complications like wound infection (3%), delayed ileus (3%), anastomotic leak (2%) and other complications (3%) with cumulative incidence of late postoperative complications of 7% in 20 years [20]. No such complications were seen in our case.

## 4 Conclusion

We report an unusual complication of Meckel's diverticulum. Challenges in preoperative diagnosis and prompt surgical treatment remain a major concern in successful management of MD. High index of suspicion for unexplained gastrointestinal bleeding, intestinal obstruction, unexplained abdominal pain, etc is needed for prompt diagnosis and management of MD. Complications of MD should be kept in mind in patients with atypical presentation. CT scan can be done to rule out complicated MD.

## Ethical approval

Not applicable.

## Sources of funding

No funding source.

## Author contribution

Author 1- Saroj Kumar Jha: Led data collection, contributed in writing the case information, introduction, discussion and final review of the manuscript. Author 2- Sharmila Ghimire: Contributed in data collection, original draft preparation and discussion. Author 3- Dinesh Prasad Koirala: Study concept, revised it critically for important intellectual content, contributed in review and editing. All the authors approved of the final version of the manuscript and agreed to be accountable for all aspects of the work ensuring questions related to the accuracy or integrity of any part of the work are appropriately investigated and resolved.

## Consent

Written informed consent was obtained from the patient for publication of this case report and accompanying images. A copy of the written consent is available for review by the Editor-in-chief of this journal on request.

## Registration of research studies

Name of the registry: Not Applicable
Unique Identifying number or registration ID:
Hyperlink to your specific registration (must be publicly accessible and will be checked):

## Guarantor

Dr. Dinesh Prasad Koirala, Tribhuvan University Teaching Hospital, Institute of Medicine, 44600 Kathmandu, Nepal. Email: koiraladinesh1@hotmail.com, Phone: +977-9804864344.

## Declaration of competing interest

No conflicts of interest.
